# Comparison of Two Different Accelerated Corneal Cross-linking Procedure Outcomes in Patients with Keratoconus

**DOI:** 10.4274/balkanmedj.galenos.2020.2019.8.45

**Published:** 2020-04-10

**Authors:** Kemal Özülken, Gözde Aksoy Aydemir, Emre Aydemir, Hasan Kızıltoprak, Erdem Yüksel

**Affiliations:** 1 Department of Ophthalmology, TOBB ETU School of Medicine, Ankara, Turkey; 2 Clinic of Ophthalmology, Adıyaman University Training and Research Hospital, Adıyaman, Turkey; 3 Clinic of Ophthalmology, Bingöl Maternity and Child Diseases Hospital, Bingöl, Turkey; 4 Department of Ophthalmology, Kastamonu University School of Medicine, Kastamanonu, Turkey

**Keywords:** Accelarated cross-linking, coma, corneal cross-linkin, cxl, higher-order aberations, keratoconus

## Abstract

**Background:**

Corneal cross-linking treatment is the unique treatment method that can cease the progression of keratoconus disease. Because of the long duration of conventional treatment, accelerated cross-linking treatment methods are being developed.

**Aims:**

To compare two different accelerated corneal cross-linking protocols in terms of postoperative visual acuity and topographic findings (higher-order aberrations and keratometry values).

**Study Design:**

Retrospective comparative study.

**Methods:**

Sixty-five eyes of 43 patients (30 men and 13 women) who underwent two different accelerated corneal cross-linking protocols (10 min, 9 mW/cm^2^ and 5 min, 18 mW/cm^2^) for progressive keratoconus were retrospectively analyzed. Patients were divided into two groups according to the accelerated corneal cross-linking treatment protocol: group 1 (10 min, 9 mW/cm^2^, 32 eyes of 21 patients) and group 2 (5 min, 18 mW/cm^2^, 33 eyes of 22 patients). Uncorrected visual acuity and best-corrected visual acuity values and topographic findings (central corneal thickness and flat and steep keratometry values) were recorded preoperatively and 6 months after corneal cross-linking treatment. High-order aberration values measured with Pentacam preoperatively and 6 months after corneal cross-linking were also recorded.

**Results:**

In both groups, a significant improvement was detected in the uncorrected visual acuity and best-corrected visual acuity levels preoperatively and 6 months postoperatively (group 1: p=0.001, p=0.001 and group 2: p=0.001, p=0.001, respectively). In addition, central corneal thickness values decreased significantly in both groups (p=0.006 and 0.001). Trefoil values showed no significant difference preoperatively and 6 months postoperatively in group 1 (p=0.160 and 0.620, respectively). In groups 1 and 2, coma values were found to decrease significantly in the 6^th^ postoperative month compared with preoperative values (p=0.001 and 0.020, respectively). There was no significant difference between preoperative and 6^th^ month postoperative horizontal and vertical trefoil values in both groups (p=0.850 and 0.140, respectively). There was no significant difference between the two groups in terms of preoperative and 6^th^ month postoperative higher-order aberrations, refractive errors, keratometry values, and uncorrected visual acuity and best-corrected visual acuity levels.

**Conclusion:**

Both accelerated corneal cross-linking procedures provide similar improvement in topographic findings, coma values and visual acuity.

Keratoconus is a progressive degenerative disease, which is usually seen bilaterally, and is characterized by the progressive thinning of the central cornea and protrusion of the corneal stroma (1). This thinning of the stroma layer in keratoconus patients results from biochemical, genetic, and environmental factors. Treatment options for keratoconus disease may include glasses, contact lenses, intracorneal segment implantation, or a combination of all these (2). However, these treatment options cannot stop the progression of keratoconus disease, and corneal transplantation may be the only option for future rehabilitation. In 1997, Spoerl et al. (3) showed that corneal cross-linking (CXL) with riboflavin and ultraviolet A (UV-A) could prevent the progression of keratoconus disease and reduce the need for keratoplasty. Corneal CXL treatment for progressive keratoconus patients increases the number of cross-linkages between the collagen fibers in the corneal stroma, making the cornea more rigid and regular (4).

The most commonly used method for CXL treatment is the conventional CXL method described by Wollensak (5). In this method, corneal iso-osmolar riboflavin solution is applied for 30 min, and then, after saturating the cornea with riboflavin, a 3 mW/cm^2^ UV-A beam is applied. The duration of treatment is about 1 h, and the cumulative UV-A dose is 5.4 J/cm^2^. Because a surgical procedure takes a long time, it may cause the corneal stroma to become more dry and thinner and increase the risk of infection (6,7). Furthermore, the long duration of the procedure makes it more difficult for the patient to endure. Also, in the centers providing intensive polyclinic service, the number of operations that can be performed on the same day can be limited.

Based on the Bunsen–Roscoe law of reciprocity, it has been proposed that it could be possible to shorten the duration of treatment using higher irradiation (8). Accordingly, it has been argued that by modifying the intensity and irradiation time, the photochemical effect will be similar. The results of experimental and clinical studies have shown that similar biological effects can be achieved with accelerated CXL treatment, which reduces the irradiation time and increases the radiation intensity (9).

Progressive distortion in keratoconus causes irregular astigmatism, progressive myopia, and increased high-order aberrations (HOAs). Therefore, attempts have been made to benefit from HOAs in the diagnosis and classification of keratoconus. Recently, CXL treatment applied to stop keratoconus disease has also been shown to regularize the optic surface and improve HOAs and have positive effects on visual functions (10). In the literature, it was seen that most of the studies investigating the effects of CXL treatment on HOAs were performed by conventional CXL treatment, or conventional CXL treatment was compared with the accelerated CXL treatment.

This study aimed to compare the effects of two different accelerated CXL treatment protocols (10 min, 9 mW/cm^2^ vs 5 min, 18 mW/cm^2^) on HOAs and best-corrected visual acuity (BCVA).

## MATERIALS AND METHODS

Patients who were diagnosed as progressive keratoconus in our clinic and underwent accelerated CXL were retrospectively analyzed. The patients were divided into two groups according to the accelerated CXL treatment protocol: group 1 (10 min, 9 mW/cm^2^) and group 2 (5 min, 18 mW/cm^2^). The following changes within 1 year were accepted as progression criteria: greater than 1 Diopter (D) increase in maximum keratometry value, visual acuity worsening due to keratoconus, more than 1 D increase in manifest astigmatism, more than 0.5 D increase in manifest spherical value or spherical equivalent, and ≥2% decrease in central corneal thickness (CCT) from baseline (11). Patients who had anterior segment surgery, an existing corneal scar, corneal trauma history, herpetic keratitis, dry eye, and autoimmune disease and those who had undergone CXL treatment and had the thinnest point of corneal thickness (less than 400 microns) on topography were excluded.

The study was approved by the local ethics committee and was conducted in accordance with the Declaration of Helsinki. All participants were given detailed information before the operation and after their written informed consent was obtained.

Demographic characteristics of the patients, preoperative and 6^th^ month postoperative 6^th^ manifest refraction values, uncorrected visual acuity (UCVA), BCVA, biomicroscopy, fundoscopy examination, keratometry values (CCT and flat vs steep keratometry values), and corneal aberrations were evaluated. The BCVA and UCVA of patients were recorded using Snellen’s chart and then converted to logMAR for statistical analysis.

Keratoconus was diagnosed with a Scheimpflug camera and Placido disk-based topography WaveLight^®^Oculyzer II (Pentacam, Germany), and the same topographic device was used in the follow-up of the patients. Topographic measurements were taken by the same technician at the same time of the day to avoid any corneal hydration differences. Furthermore, the measurements were repeated until an optimum measurement was obtained.

Aberration measurements and corneal topography were analyzed using the WaveLight^®^Oculyzer II. Total corneal HOAs including a coma, horizontal and vertical trefoil, spherical aberration, total HOA, and Q value (corneal asphericity) in the Zernike analysis were recorded. Total corneal aberrations, calculated from the elevation values by the Pentacam software, were evaluated in a 6.0-mm diameter central area with respect to the pupil center in a dark environment, and the pupil was not dilated.

### Surgical technique

A single experienced surgeon (K.O.) performed all surgeries. Under sterile conditions at the operating room, following draping, a 0.5% proparacaine HCl ophthalmic solution (Alcaine, Alcon Inc., Hünenberg, Switzerland) was instilled into the eyes. A 9.0-mm diameter corneal epithelium was peeled after stripping with the help of a spatula, and then, a 0.1% riboflavin solution (0.1% solution VibeX, Avedro Inc., Waltham, Massachusetts, USA) was applied to the cornea every 3 min for half an hour. In group 1, after the control of the riboflavin solution penetrated the entire cornea with a surgical microscope, the cornea was irradiated with UV-A light at a wavelength of 370 nm and 9 mW/cm^2^ for 10 min. In group 2, the cornea was irradiated with an 18 mW/cm^2^ UV-A light at 370 nm for 5 min. In both groups, a drop of riboflavin solution was applied every 3 min also during the irradiation period. After the procedure, all patients were fitted with soft contact lenses (Lotrafilcon B, Air Optix Hydraglyde, Alcon Laboratories Inc.) with a diameter of 14.0 mm, base curve of 8.6, and a maximum of 140 barrers. Postoperative treatment included 0.5% moxifloxacin hydrochloride and 0.1% fluorometholone eye drops four times a day. Patients were monitored daily until the corneal epithelium recovered, and the contact lenses were removed after epithelial healing. Follow-up examinations of the patients were made in the second week, first month, third month, and sixth month.

### Statistical analysis

Descriptive data were presented as the means ± standard deviations, frequency distributions, and percentages. The Shapiro-Wilk test was used to assess the conformity of the data to normal distribution. The Wilcoxon test was used to analyze data that were not normally distributed in dependent groups and the Mann-Whitney U test for non-normal distributed data in nondependent groups. Data were analyzed using SPSS Windows 20.0 software (IBM, Armonk, New York, USA). A value of p<0.05 was considered statistically significant. The results of a priori power analysis via power and sample size calculation software, version 11, showed the need to enroll at least 30 eyes in each group. Therefore, 32 eyes were included in group 1 and 33 eyes in group 2; the power of the study was found to be 82.3%. The primary outcomes of our study are visual acuity, corneal keratometry, refractive error, higher-order aberration values, and their changes preoperatively and 6 months postoperatively. From the references, articles using variables similar to our study were selected, and the effect size and minimum sample size were calculated. The effect size was accepted as 0.60, type 1 error rate as 0.05, and type 2 error rate as 0.20. The primary outcomes are UCVA, BCVA, corneal keratometry, aberration values, and their changes preoperatively and 6 months postoperatively.

## RESULTS

The study included 65 eyes of 43 patients (30 men and 13 women). Group 1 comprised 32 eyes of 21 patients (17 men and 4 women) with a mean age of 23.23±4.21 years (16-33 years). Group 2 comprised 33 eyes of 22 patients (13 men and 9 women) with a mean age of 23.90±4.44 years (17-34 years). There was no significant difference between the two groups in terms of preoperative keratometry, HOAs, visual acuity, and spherical cylindrical values (p>0.05 each).

### Visual acuity and refraction results

Preoperative values were compared between groups. No significant differences were detected (p=0.008). The visual acuity and refraction values of the patients before and at 6 months after CXL treatment are shown in Table 1. In Group 1, a significant difference was found between UCVA and BCVA before and 6 months after the CXL treatment (p=0.001 each). Refractive examination revealed a significant decrease in spherical and cylindrical values at 6 months after treatment (p=0.001 each). In group 2, there was a significant difference in UCVA and BCVA before and 6 months after the treatment (p=0.001 each). The refractive examination at 6 months showed a significant improvement in spherical and cylindrical values (p=0.001 each). When the visual acuity levels of two groups were compared preoperatively and 6 months postoperatively, no significant difference was found between the two groups (p=0.123). Changes from the pre-post treatment in UCVA and BCVA in each group showed no statistically significant difference between groups (p>0.05 each), which is shown in Table 2.

### Topography results

Preoperative corneal topography results were compared between groups. No significant differences were detected (p=0.128). The corneal topography results of the patients before and 6 months after treatment are shown in Table 3. In group 1, a statistically significant decrease was determined in steep and flat keratometry values at 6 months after CXL treatment compared with the pretreatment values (p=0.001 each). The thinnest corneal thickness decreased by approximately 6 µm (p=0.006). In group 2, flat and steep keratometry values at 6 months after the CXL treatment decreased by 0.2 and 0.5 D, respectively, and the differences were statistically significant (p=0.030 and 0.001, respectively). A decrease of 8.5 µm was found to be statistically significant in the thinnest corneal thickness (p=0.001). When topography results of two groups were compared preoperatively and 6 months postoperatively, no significant difference was found between the two groups (p=0.092). Changes from the pre-post treatment in topography results in each group showed no statistically significant difference between groups (p>0.05 each) as shown in Table 4.

### HOA results

**Figure 1:**
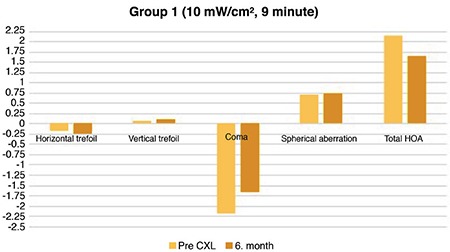
Precorneal cross-linking and postcorneal cross-linking 6^th^ month higher-order aberration values in Group 1 (10 mW/cm^2^, 9 min).

Preoperative HOA results were compared between groups. No significant differences were detected (p=0.487). The HOA results of the patients before and 6 months after treatment are shown in Table 5 There was no significant difference in the horizontal and vertical trefoil values of the patients in group 1 before and 6 months after treatment (p=0.160 and 0.620, respectively). A significant difference was observed in a coma before and after CXL treatment in group 1 (p=0.001). A significant difference was observed in the total HOA (p=0.001), whereas no significant difference was observed in spherical aberration in group 1 before and 6 months after treatment (p=0.420) (Figure 1).

**Figure 2:**
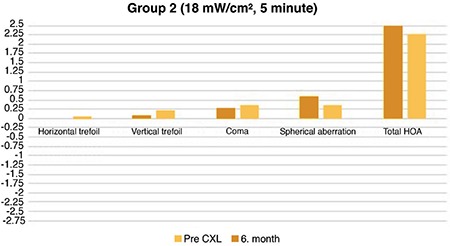
Precorneal cross-linking and postcorneal cross-linking 6^th^ month higher-order aberration values in group 2 (18 mW/cm^2^, 5 min).

In group 2, no significant difference was observed in the horizontal and vertical trefoil values before and 6 months after CXL treatment (p=0.850 and 0.140, respectively). There was a significant difference in a coma (p=0.020). Although there was no significant difference in spherical aberration (p=0.060), a significant difference was observed in total HOA (p=0.001) (Figure 2).

When HOA values of two groups were compared preoperatively and 6 months postoperatively, no significant difference was found between the two groups (p=0.140). Changes from the pre-post treatment in HOA values in each group showed no statistically significant difference between groups (p>0.05 each) as shown in Table 6.

## DISCUSSION

The present study investigated the effects of two different accelerated CXL treatments on visual acuity and topographic parameters. It is known that structural changes in the cornea can decrease visual quality by increasing HOAs.

Ocular aberrations are divided into two groups: monochromatic and chromatic aberrations. Monochromatic aberrations are divided into two subgroups, namely, low-order aberrations (spherical and cylindrical refractive defects) and HOAs (spherical aberration, secondary astigmatism, coma, trefoil, quadrofoil, tetrafoil, and pentafoil) (10). Since ocular aberrations are a major cause of visual impairment in keratoconus, (8,9,10) the difference of HOAs between the two different accelerated CXL protocols was evaluated in this study. A

3 mW/cm^2^ 30-min conventional CXL treatment has been shown to stop keratoconus disease and improve vision, topographic parameters, and HOAs (11). However, in conventional CXL treatment, 30 min of riboflavin and 30 min of UV-A administration for a total of 1 h can be very demanding for both the patient and the surgeon. Moreover, occupying the operating room for about 1 h reduces the total number of operations that can be performed, thereby reducing efficiency (12). In addition, it may be risky for infection to expose the cornea for 1 h after corneal epithelial peeling (13). Therefore, based on the Bunsen–Roscoe reciprocity law, the same biological effect can be achieved with higher irradiation for a much shorter time, and this has been described as accelerated CXL treatments (14). The shortening of the total treatment time by providing a much higher irradiance was described as accelerated CXL (15). Accelerated CXL was started to be used in ophthalmology practice as it provided both patient and doctor a high degree of convenience by shortening the treatment period (16). However, the biggest concern in accelerating CXL was whether it was as effective as standard treatment. In ex vivo studies on corneal biomechanical properties, Wernli et al. (17) demonstrated that Bunsen–Roscoe reciprocity law was valid for irradiance values up to 40–45 mW/cm^2^ (treatment time of less than 2 min) and that porcine corneas up to these values are acceptable corneal stiffness. In addition, Hammer et al. (7) observed that 9 mW/cm^2^ treatment showed similar corneal stiffness with the standard treatment, but values above this (18 mW/cm^2^) were not different from the control group. They considered this because oxygen consumption in accelerated procedures is very fast, and oxygen in the environment is exhausted very quickly and cannot diffuse sufficiently into the cornea (7). As is known, oxygen and oxygen radicals are responsible for the formation of a type 2 reaction, which plays an important role in the initiation and continuation of the CXL process (18). However, Viciguerra (19) stated that the smoothing effect of CXL on the cornea was not directly related to the increase in best spectacle-corrected visual acuity and the increase in corneal biomechanics. These results have encouraged many surgeons to accelerated CXL, and many studies have been conducted (20,21,22).

In their study comparing accelerated (5 min, 18 mW, 5.4 J/cm) and standard (30 min, 30 mW, 5.4 J/cm) CXL treatments, Kato et al. (23) reported a significant decrease in keratometry values in both methods at the end of 1 year, and no difference was found between the two methods. Likewise, Yildirim et al. (24) observed that topographic and refractive changes improved significantly and similarly at the end of 12 months after both accelerated and standard CXL treatments. In our study, accelerated CXL treatment in two different protocols showed a significant increase in visual acuity and a significant decrease in spherical and cylindrical values and K1 and K2 values. We did not observe a significant difference between these two protocols. Despite studies indicating that there was no difference between the two methods in terms of topographic improvement, the study of Kirgiz et al. (20) that compared accelerated CXL of 10 min (9 mW) and 5 min (18 mW) found that 10 min CXL was better in terms of topographic recovery. Choi et al. (21) found that the topographic improvement was better at 3 mW irradiance, even if it increased 30 mW irradiance and 6.6 J/cm total energy. When Hashemi et al. (22) compared the long-term results of standard (3 mW) and accelerated (18 mW) CXL, they found that standard treatment significantly improved topographic flattening and refractive correction. The possible explanation of obtaining different results in studies comparing accelerated and standard CXL can be patients’ age, various devices used, and different preoperative keratometry and visual values. However, the reason for less topographic flattening with accelerated CXL was interpreted as the appearance of a more superficial demarcation line with accelerated CXL (21,22). This is thought to be because the oxygen in the environment is exhausted at high energy and cannot diffuse sufficiently into the cornea (18). In our study, we did not evaluate the demarcation line with OCT. This can be considered as the weakness of our study.

In keratoconus disease, visual impairment is caused by high irregular astigmatism as well as increased HOAs (25). Coma-like aberrations in keratoconus patients are known to be much higher than in normal eyes (26). In addition to stopping keratoconus disease, CXL treatment leads to topographic changes, increases visual acuity, and improves visual quality by lowering HOAs (8).

In this study, the HOA values of the two groups were examined. When the values of patients in group 1 (10 min, 9 mW/cm^2^) and group 2 (5 min, 18 mW/cm^2^) before and after CXL were compared, a significant decrease was observed only in coma and total HOA. Although visual acuity and topography findings improved, similar findings were not obtained in aberrations. Further studies are needed to elucidate the effects of the changes in HOAs after CXL treatment on visual function and contrast sensitivity, as well as possible relationships with low-contrast visual acuity.

In contrast to the improvement in visual acuity and K1 and K2 values, one of the reasons for not having similar results in HOAs may be the difference in the preoperative topographic cone location in patients. Unlike the current study, Greenstein et al. (27) found a significant reduction in HOAs in the 1^st^ year follow-up of the standard CXL protocol (3 mW/30 min) for the keratoconus and post-LASIK ectasia. Unlike our study, this study had a long follow-up period, and standard CXL protocol was applied. Kocamış et al. (28) applied the standard CXL protocol to 37 keratoconus patients and evaluated the HOAs in the 1^st^, 3^rd^, 6^th^, 12^th^, and 18^th^ month. In the early follow-up period, especially in a vertical coma, they found a significant decrease in HOAs and total corneal aberrations in the 18^th^ month examination. Caporossi et al. (29) found a statistically significant decrease in HOAs after 48 months of follow-up. Baumeister et al. (30) examined the short-term effects of CXL therapy, and in the 6^th^ month, a decrease in a coma aberration was observed, and no statistically significant decrease was found in total HOAs. Kirgiz et al. (20) also reported that coma-like HOAs were significantly lower in the 9 mW group than in the 18 mW group. In the current study, the short follow-up period and the application of two different accelerated CXL protocols instead of standard CXL protocol may have been the reason that significant difference only observed in a coma and total HOA in groups 1 and 2.

The lack of aberrometer for the measurement of HOAs, the low number of patients, and the lack of a control group with the standard CXL protocol treatment were among the factors limiting this study. In addition, the comparison of patients with data only in the 6^th^ month after CXL is another missing side of our study.

Another limitation of the study is the lack of endothelial cell density measurement, so there were no data on the effects on the corneal endothelium of accelerated CXL treatment applied with higher energy. However, a retrospective examination of the patients revealed no postoperative complications such as corneal decompensation, cataract, or chronic epithelial defect due to endothelial loss.

Future studies are needed to elucidate the impact of changing HOAs on visual function after CXL treatment. These studies can also investigate the possible relationships of HOAs with contrast sensitivity and low-contrast visual acuity.

In conclusion, despite the difference of CXL treatment protocols, similar findings were obtained in both groups. Although the effect on HOAs cannot be demonstrated, there is a need for further studies with larger patient groups and longer follow-up period to be able to re-evaluate and draw more definitive conclusions.

## Figures and Tables

**Table 1:**
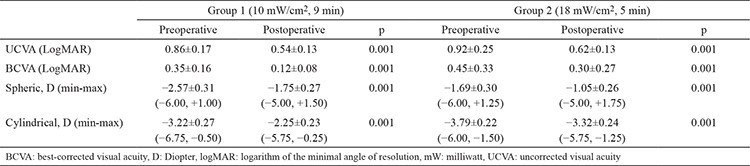
Preoperative/postoperative visual acuity and refraction values

**Table 2:**
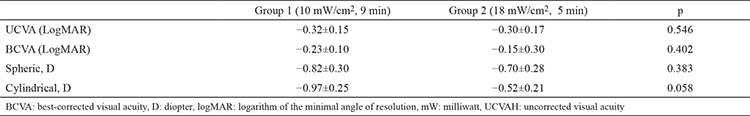
Changes between preoperative and postoperative visual acuity and refraction values between groups

**Table 3:**
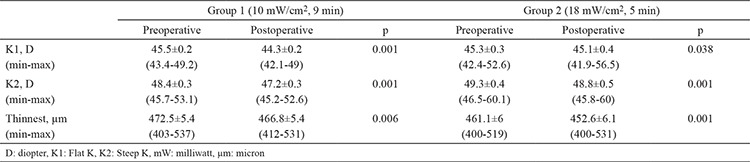
Preoperative and postoperative topographic values

**Table 4:**
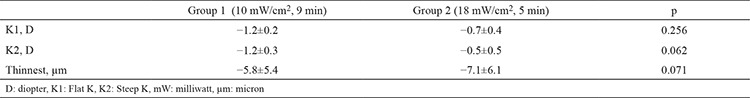
Changes between preoperative and postoperative topographic values between groups

**Table 5:**
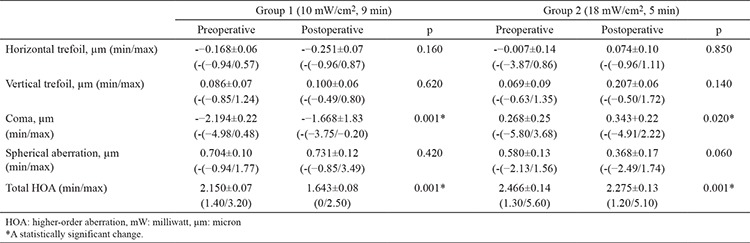
Preoperative and postoperative high-order aberration values

**Table 6:**
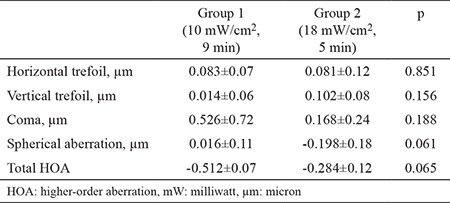
Changes between preoperative and postoperative high order aberration values between groups
